# Chitosan Acts as a Sustainable Strategy for Integrated Management of Root-Knot Nematodes (*Meloidogyne* spp.) in Cherry Tomato

**DOI:** 10.3390/plants15020256

**Published:** 2026-01-14

**Authors:** Carolina González-Cardona, Juan Camilo Orrego-Cardona, Alejandro Ospina-Gutiérrez, Claudia Nohemy Montoya-Estrada, Jairo Eduardo Leguizamón-Caycedo, Mauricio Soto-Suárez, Alejandro Hurtado-Salazar, Nelson Ceballos-Aguirre

**Affiliations:** 1 Doctorado en Ciencias Agrarias, Facultad de Ciencias Agropecuarias, Universidad de Caldas, Calle 65 No. 26-10, Manizales 170003, Caldas, Colombia; jairolegui1963@hotmail.com (J.E.L.-C.); alejandro.salazar@ucaldas.edu.co (A.H.-S.); nelson.ceballos@ucaldas.edu.co (N.C.-A.); 2 Programa de Ingeniería Agronómica, Facultad de Ciencias Agropecuarias, Universidad de Caldas, Calle 65 No. 26-10, Manizales 170003, Caldas, Colombia; juancamilo.orregocardona@gmail.com (J.C.O.-C.); alejo281099@hotmail.com (A.O.-G.); 3 Instituto de Investigaciones en Microbiología y Biotecnología Agroindustrial, Universidad Católica de Manizales, Carrera 23 N° 60-63, Manizales 170003, Caldas, Colombia; cmontoya@ucm.edu.co; 4 Corporación Colombiana de Investigación Agropecuaria-AGROSAVIA, Km 14 Vía, Mosquera, Bogotá, Mosquera 250047, Cundinamarca, Colombia; msoto@agrosavia.co

**Keywords:** nematode, chitin, edaphic application, foliar application, *Solanum lycopersicum*

## Abstract

Root-knot nematodes (*Meloidogyne* spp., RKN) penetrate the roots of plants, blocking the flow of water and nutrients, preventing plant development, and causing losses of up to 68% in production. Its management is limited by the low availability of genetically resistant materials, the inefficient use of biological controllers, and the high risk of environmental contamination from the application of pesticides. The aim of this study was to contribute to the integrated management of (RKN) through the use of chitosan. A completely randomized experimental design was used in a factorial arrangement with two applications (foliar or edaphic), two cherry tomato genotypes (IAC1687 and LA2076), and eight treatments (three concentrations of chitosan (1.5–2.0–2.5 mg/mL), commercial controls and absolute controls). The yield and nematode population components were evaluated. The cherry tomato (IAC1687) obtained the greatest yield, with 33.517.1 kg/ha and an 85% reduction in the nematode population with the application of 2.5 mg/mL of chitosan to the soil. Chitosan improved the yield components of the evaluated cultivars and reduced nematode populations, suggesting that it can be a sustainable alternative in commercial production systems, as it can help reduce the use of chemical pesticides and improve health and crop productivity. As a limitation of this study, the use of acetic acid as a solvent for chitosan potentially interfered with the results associated with the nematode population, increasing bias and imprecision as there was no blockage due to light, temperature, or irrigation. Therefore, we suggest that future research explores alternative solvents to elucidate the mechanism of action or response of chitosan.

## 1. Introduction

The tomato, *Solanum lycopersicum*, is one of the most socioeconomically important horticultural crops worldwide, providing vitamins, folic acid, potassium, calcium, magnesium, iron, phosphorus, and phytochemicals such as lycopene and carotenoids [1]. It ranks first in vegetable production, with a harvested area of 5.030.545 hectares and a yield of 359.337 tons per hectare in 2022. Of this production, 576.733 t were produced in Colombia on 8.478 cultivated hectares [2].

The main limiting factors for production are diseases caused by pathogens such as *Phytophthora infestans*, *Fusarium oxysporum*, and plant parasitic nematodes (PPNs) of the genus *Meloidogyne* spp., which cause galls on the roots, restricting the movement of water and nutrients to the plant [3] and leading to production losses of up to 68% [4]. Most management practices gradually reduce nematode numbers over periods ranging from weeks to months but none of the available strategies are entirely effective; therefore, multiple methods must be integrated to help reduce nematode populations and improve production [5].

Molecules such as chitosan are promising for agricultural use due to their ability to reduce the incidence and severity of various plant diseases by inducing resistance through the activation of certain pathogenesis-related enzymes [6] and the production of reactive oxygen species (ROS), which is a conserved defense response in plants and is induced in the early stages of infection [7]. Chitosan is a biopolymer derived from the deacetylation of chitin, found in the cell walls of Zygomycetes, some green algae, yeasts and in the exoskeletons of insects and crustaceans. It is the second most abundant renewable carbon source in the world after lignocellulose [8,9]. Its effectiveness depends on the target organism, molecular weight, concentration, degree of deacetylation, and type of chitosan, among other factors [10,11,12].

Chitosan has a nematicidal function that is highly useful in agriculture. reducing nematode populations, affecting their morphophysiological characteristics and mitigating the degree of damage or severity in plants [13,14,15]. Khalil et al. [12,16] evaluated the nematicidal activity of four molecular weights of chitosan on *M. incognita* under in vitro conditions and in greenhouse tomato seedlings, finding that nematicidal activity increases as molecular weight decreases. suggesting that low molecular weight chitosan can serve as an effective nematicide.

The investigation of chitosan as an alternative strategy for nematode management represents a promising approach to replace conventional chemical nematicides, which are frequently associated with environmental contamination and risks to human health. Chitosan, a natural polysaccharide obtained from chitin present in crustacean exoskeletons, is biodegradable and exhibits a lower environmental impact compared to synthetic chemicals. Furthermore, chitosan demonstrates a dual mode of action: it exerts direct effects on nematodes and enhances plant defense mechanisms through the induction of systemic resistance. Its application may also contribute to reducing dependence on costly and highly regulated chemical inputs, while promoting the valorization of by-products from the fishing industry, thereby supporting added value generation and the principles of a circular economy. Therefore, the aim of this study was to contribute to the integrated management of RKN through the use of different concentrations of chitosan as an alternative to improve plant health and yield, reduce economic losses, and mitigate the environmental impact of agrochemicals.

## 2. Results

### 2.1. Chitosan Soil Application in Tomato Cherry Genotypes IAC1687 and LA2076

In the IAC1687 variety, the highest production per plant was observed in the CK control treatment, with 1608.8 g per plant, corresponding to a yield of 33,517.1 kg/ha. This yield did not show statistically significant differences compared to treatments Q2.5 and Q2.0, which achieved yields of 24,575.8 kg/ha and 23,860.5 kg/ha, and production per plant of 1179.6 g and 1145.3 g, respectively. Regarding the nematode population variable, CK also resulted in the lowest population, with 1842 eggs per 100 g of roots, followed by Q2.5 with 3898 eggs and Q2.0 with 5384 eggs. In contrast, the AN treatment showed a population of 26,290 eggs per 100 g of roots. This represents a population reduction of 93% for CK, 85% for Q2.5, and 80% for Q2.0 compared to AN. The Q1.5 dose showed a lower yield of 13,233.1 kg/ha and a higher nematode population of 10,670 eggs per 100 g of roots, corresponding to a 59% reduction compared to AN (Table 1).

Acetic acid resulted in a 90% reduction in nematode population compared to the AN treatment; however, this difference was not statistically significant when compared to the other treatments. Although acetic acid had a positive effect on nematode suppression, its impact on yield was negative—showing a 59% reduction compared to CK, 44% compared to Q2.5, and 42% compared to Q2.0. These findings suggest that, despite its effectiveness in reducing nematode populations, acetic acid adversely affects plant productivity, a drawback not observed when it is used in the formulation of chitosan (Table 1).

In the LA2076 variety, no statistically significant differences were observed in the nematode population. However, the Q2.5 treatment achieved the greatest reduction in nematode population, with a 71% decrease compared to the AN treatment, which recorded the highest count (23,215 eggs per 100 g of roots). Despite this reduction, the yield showed significant differences when compared to the treatments involving chitosan application (Table 1).

The LA2076 variety exhibited higher yields under treatments A and AN, producing 27,389.1 kg/ha and 29,118.9 kg/ha, respectively. No statistically significant differences were observed when compared to treatments Q2.5, CK, and CQ, which yielded 23,545.0 kg/ha, 23,448.9 kg/ha, and 21,526.9 kg/ha, respectively.

Regarding nematode populations, LA2076 showed similar results to IAC1687. The greatest reduction was observed under treatment CK, with a 73% decrease compared to AN, which had the highest nematode population (23,215 eggs/100 g of roots). Treatments Q2.5 and Q2.0 followed with reductions of 71% and 68%, respectively.

In LA2076, treatment AA resulted in an 84% reduction in nematode population compared to AN. However, its yield was 6439 kg/ha lower than that obtained with the chitosan-based treatment Q2.5, which had the highest yield. This difference is statistically significant and could have economic implications for farmers (Table 1).

A significant difference in yield was observed among the cherry accessions, with IAC1687 outperforming LA2076. This suggests that IAC1687 may be better adapted to the agroclimatic conditions of the study area. Additionally, this accession demonstrated a notable contribution to nematode population reduction, attributed to the application of the chitosan molecule and the potential efficacy of acetic acid as a base in its formulation. These findings highlight the integration of effective management strategies (Table 1).

The susceptibility of *M. incognita* to experimental treatments was evaluated using the Reproduction Index (R.I.), with the nematode-infested control (AN) established as the baseline (100%). All evaluated chitosan concentrations significantly inhibited nematode reproduction in the roots of IAC1687, exhibiting a clear dose-dependent effect. The R.I. progressively decreased from 40.59% at the lowest concentration (Q1.5) to 14.83% at the highest (Q2.5), which represents an 85.17% reduction relative to the control. The commercial chitosan control (CK) and the chemical standard (CQ, fluopyram) confirmed the range of efficacy, yielding R.I. values of 7.01% and a near-total inhibition of 0.20%, respectively. The acetic acid control (AA) also resulted in a marked reduction in reproduction, reaching an R.I. of 10.07%, a result that must be considered during the interpretation of chitosan’s true mode of action. Evaluation of the LA2076 cherry tomato variety also demonstrated significant inhibition of *M. incognita* reproduction in all chitosan treatments, confirming the compound’s overall efficacy. The Reproduction Index (RI) continued to show clear dose-dependent inhibition, although overall efficacy was slightly lower compared to IAC1687. The RI for LA2076 decreased from 39.78% with Q1.5 to 29.33% with the highest concentration (Q2.5), corresponding to a 70.67% reduction compared to the control (AN, 100%). Commercial control treatments maintained their high efficacy, and the chemical standard (CQ) resulted in almost complete inhibition (RI of 0.37%) (Table 1).

### 2.2. Chitosan Foliar Application in Tomato Cherry Genotypes IAC1687 and LA2076

In IAC1687, the highest production per plant following foliar application of chitosan was observed in the Q2.0 treatment, with 1287.7 g/plant, resulting in a yield of 26,827.3 kg/ha. No statistically significant differences were found when compared to treatments Q1.5 and CK, which yielded 24,837.7 kg/ha and 23,425.7 kg/ha, with per-plant productions of 1192.2 g and 1124.4 g, respectively. Regarding nematode populations, Q1.5 resulted in the lowest count, with 2223 eggs per 100 g of roots, followed by Q2.0 with 3450 eggs and Q2.5 with 5269 eggs. In contrast, the AN treatment showed the highest nematode population, with 26,290 eggs per 100 g of roots. These results correspond to population reductions of 92% for Q1.5 87% for Q2.0 and 80% for Q2.5 (Table 2).

In the soil application, the Q1.5 dose resulted in a low yield of 13,233.1 kg/ha and a high nematode population of 10,670 eggs per 100 g of roots, representing a 59% reduction compared to the AN treatment (Table 1). However, when the same dose was applied foliarly, both yield and nematode control improved significantly. The yield increased by 11,604.6 kg/ha, and the nematode population decreased by over 8000 eggs per 100 g of roots, corresponding to a 92% reduction compared to AN (Table 2).

In LA2076, a similar trend was observed for the Q1.5 treatment in terms of nematode population, with the lowest count recorded at 4049 eggs per 100 g of roots. This represents an 83% reduction compared to AN (Table 2), and it outperformed the soil application for this genotype (Table 1).

In LA2076, the highest production per plant following foliar application of chitosan was observed in the Q2.0 treatment, with 1420.7 g/plant, resulting in a yield of 29,599.4 kg/ha. No statistically significant differences were found when compared to Q1.5, which yielded 29,503.3 kg/ha and 1416.1 g/plant. Regarding nematode populations, Q1.5 resulted in the lowest count, with 4049 eggs per 100 g of roots, followed by Q2.0 with 4524 eggs and Q2.5 with 7980 eggs. In contrast, the AN treatment showed the highest nematode population, with 26,290 eggs per 100 g of roots. These results represent population reductions of 83% for Q1.5, 81% for Q2.0, and 63% for Q2.5 (Table 2).

When we compared the response of the nematode population per 100 g of roots by evaluating only the 1.5, 2.0, and 2.5 mg/mL chitosan treatments, we found that the highest population of nematodes was registered for the 1.5 mg/mL chitosan applied edaphically in the IAC1687 and LA2076 genotypes with 10.670 and 9.235 individuals, respectively. The lowest populations were recorded for the 1.5 mg/mL chitosan applied at the foliar level in the IAC1687 genotype. The treatments with fluopyram (CQ) and acetic acid (AA) showed the most effective results in reducing nematode populations across all evaluated genotypes. For CQ, the reductions were 98% in IAC1687, 96% in LA2076. Similarly, AA achieved reductions of 90% in IAC1687, 84% in LA2076 (Table 1 and Table 2).

Although both treatments demonstrated strong nematode suppression, they were associated with a substantial decrease in crop yield (kg/ha). In IAC1687, the CQ treatment resulted in a 55% lower yield compared to the CK treatment, which had the highest production, while the AA treatment showed a 59% reduction in yield relative to the same control. In LA2076, CQ and AA treatments yielded 26% and 41% less, respectively, than the AN treatment, which recorded the highest yield for this genotype.

The efficacy of foliar chitosan application was also evaluated by measuring the Reproduction Index (RI), establishing a baseline value of 100% in the nematode-infested control (AN). Unlike the soil treatments, the foliar application showed an inversely dose-dependent relationship in nematode reproduction. For IAC1687, the lowest concentration (Q1.5) was the most effective, limiting the RI to only 8.46%, indicating a 91.54% reduction in nematode multiplication. Efficacy decreased significantly with increasing doses, with RI values rising to 13.12% (Q2.0) and 20.04% (Q2.5). The commercial control (CK) showed a final RI of 28.11%. Similarly, in the LA2076 variety, the lowest concentration (Q1.5) achieved the greatest inhibition (RI of 17.44%). The inhibitory effect decreased markedly at higher concentrations, with Q2.5 (RI of 34.37%) and CK (RI of 32.38%) being the least effective treatments. This consistent trend suggests that the main mechanism of action following foliar application is the systemic induction of the host plant’s defense mechanisms, which are optimally activated at lower concentrations, severely limiting the nematode’s reproductive capacity (Table 2).

## 3. Discussion

### 3.1. Chitosan Foliar and Edaphic Application in Tomato

That low applications of chitosan at the foliar level possibly induce resistance in the plant, resulting in a reduction in the nematode population. When these concentrations increase and are applied edaphically, fewer nematodes are observed. This study found differences between tomato genotypes and methods of applying the treatments. According to Rodríguez [17], the effect of chitosan varies according to the morpho-physiological characteristics of the species. Additionally, Mota [18] indicated that the application of chitosan to tomato plants infested with *M. javanica* reduced the J2/pl by 83%. The chitosan molecule could be showing a hormetic effect in this case. Yu [19] has shown that these substances can have a hormetic effect in *Caenorhabditis elegans*; at low doses, they can stimulate beneficial processes, but at very high doses, they generate a decline in the treatment’s effectiveness. The hormetic effect has been reported in several agents (metals, plastics, chemicals, among others), as well as in environmental factors such as stress and temperature changes.

### 3.2. Chitosan in the Formation of Galls and Reproduction Index of Nematodes

In the management of nematodes in tomato, El-Sayed [20] evaluated both low- and high-molecular-weight chitosan in tomato crops infested with *M. javanica* under greenhouse conditions. He reported that at a low molecular weight of 2%, there was a 92% reduction in root nodules, 92% reduction in root egg masses, 77% reduction in J2, and 92% reduction in females. Additionally, a positive genetic effect can be obtained, where the plants reach production despite the population or attack of the disease. Studies on nematodes in promising tomato genotypes show that the IAC1687 genotype presented the resistance allele of the *Mi-1* gene and was classified as moderately resistant at a density of 1000 individuals per plant [21,22].

The use of chitosan to reduce nematode populations has been promising. Foliar applications decreased the J2 of *M. incognita* by between 46.62% and 55.23% [16]. However, evaluated tomato crop liquid chitosan at 0.5%, 1%, and 2%, showing the best response compared to a chemical treatment with fluopyram, where this treatment obtained the best results, with 94.3% control over nodulation, 93.5% control over egg mass, and 87.6% control over J2 in the soil [23]. These same authors [16] evaluated the foliar and edaphic application of low-molecular-weight chitosan to tomato plants and reported a decrease in the population density of *M. incognita* alone or in the presence of tobacco mosaic virus (TMV) between 45.89% and 66.61%, and the density of the roots decreased between 10.63% and 67.87%. For foliar applications, a decrease in J2 was observed between 46.62% and 55.23%, and for drench applications, a decrease in J2 between 58.28% and 64.5% was obtained.

The nematicidal activity of chitosan increases as its molecular weight decreases. Low-molecular-weight chitosan is a natural nematicide because the positive charge of chitosan acts on the negative layer of *Meloidogyne* eggs, altering the permeability of the cell membrane and causing death due to the loss of essential materials such as proteins and intracellular constituents [12]. Demonstrated that the application of chitosan to soil controls *Meloidogyne* spp. [23,24]. Organic materials, including chitin, can change soil physicochemical properties, release nematicidal compounds such as organic acids and nitrogen compounds (NH_3_), and can induce plant resistance by increasing antagonistic microorganisms in the soil, resulting in the suppression of nematode populations.

Analysis of the results indicated that although 1% acetic acid at a pH of 5.6 applied to the plants can reduce nematodes compared to the control treatments, the average yields were not the same. When acetic acid was applied, there was a reduction in the nematode population per 100 g of roots. However, the average production remained below the best treatments. This may be associated with the herbicidal characteristics [25,26,27]. Acetic acid has nematicidal properties, inhibiting the hatching of eggs and the release of juveniles in *M. javanica*. It also negatively impacts the abundance of free-living nematodes [28], and when used in mixture with maleic acid, it has been shown to induce 100% mortality in J2 of *M. incognita* [29].

Although CQ and AA present better responses in the percentage reduction in *Meloidogyne*, the production and yield of the plants are not as good compared to chitosan-based treatments, showing values below 50%. CQ exhibits a population reduction greater than 90% in most genotypes, but in vitro evaluations corroborated the nematostatic effect of the molecule, similar to what was reported by Schleker [30], suggesting a recovery of the microorganisms to continue their parasitic process.

However, the nematicidal effect could be attributed to both acetic acid and chitosan or to a synergistic effect, which may vary depending on factors such as genotype, concentration, application method, nematode species or environmental conditions.

Acetic acid can alter the cell membranes and metabolic processes of nematodes, causing their malformation and mortality [27]. For its part, the positively charged amino groups of chitosan can bind to negatively charged components in nematodes, interfering with their physiology, it can also alter the cuticle and cause osmotic stress or physical damage [31,32].

From a nutritional point of view, plants treated with chitosan benefit as described by several authors. The concentration of chitosan applied influences nutrient uptake by plant roots and stimulates nutrient biosynthesis and defense mechanisms through its biostimulant effect [33]. The application of chitosan increases the production of defensive enzymes, involving biochemical and physiological processes that consume energy and photoassimilates from plants [33,34]. Chitinase and β-1,3-glucanase are two proteins that contribute to plant protection via resistance mechanisms [34]. In addition to having a nutritional benefit, there is also an increase in the quality of fruits.

In *Capsicum annuum*, plants sprayed with chitosan on both leaves and fruits exhibited an increase in fruit weight, fruit diameter, and yield compared to the control, with values such as 20% more leaves, 11% greater plant height, and 34.64% greater fruit and fruit weight [35]. With the foliar application of 5 mL/L chitosan to mango leaves and fruits, 37 more fruits per tree and 3 g more fruit weight were obtained, indicating a yield of 44.1 kg/tree compared to the control treatment (32.6 kg/tree) [35,36]. Concentration of 1000 mg/L foliar chitosan in tomato plants, 25 fruits per plant, a fruit weight of 171 g, and a yield of 6 t/ha were obtained, while the control without application yielded 18 fruits per plant, 125 g of fruit weight, and a yield of 3 t/ha [36].

The interactions among genotypes IAC1687 and LA2076, with different concentrations of low-molecular-weight chitosan applied both via foliar and edaphic methods had positive effects on yield variables and nematicidal effect against *Meloidogyne* spp. However, these results varied according to the variable and genotype evaluated.

## 4. Materials and Methods

### 4.1. Location

The experiment was carried out at Montelindo Farm (5.075442 N, −75.673229 W), located in the village of Santágueda, municipality of Palestina (Caldas, Colombia). The site is situated at an altitude of 1010 m a.s.l., with an average temperature of 22.8 °C, annual rainfall of 2200 mm, relative humidity of 76%, and 2010 h of sunshine per year. Laboratory processes, including obtaining and identifying the inoculum, preparing the chitosan, and sample processing, were conducted in the Phytopathology Laboratory of the Universidad de Caldas, Caldas, Colombia.

### 4.2. Plant Material

Seeds of two cherry tomato (*Solanum lycopersicum* var. ceraciforme) accessions, IAC1687 from the Agronomic Institute of Campinas, Campinas, SP, Brazil, and LA2076 from the seed collection of the Tomato Resources Institute of California, Davis, CA, USA, were selected for their tolerance to nematodes [21,22].

Cherry tomato has an average fruit weight ranging from 10 to 30 g, with the number of fruits per cluster ranging from 15 to 50 [37]. The number of flowers per cluster varied from 12 to 117, and the number of clusters per plant ranged from 10 to 13 [37]. Therefore, the expected yields are variable. Similarly, nutritional characteristics vary in terms of vitamin C, lycopene, and β-carotene contents, with values for cherry tomatoes of 57 mg/100 gpf, 10 mg/100 gpf, and 3.0 µg/g, respectively.

### 4.3. Plant Distribution

The seeds were sown in 128-locule planting trays filled with “Sphagnum” peat substrate for twenty days before transplanting. Transplanting was carried out into 10 kg plastic bags using soil previously disinfected by applying dazomet (500 g/m^2^) and solarization. The plants were grown under a closed macro-tunnel greenhouse system to ensure semi-controlled conditions during the experiment. The plants were spaced 1.2 m apart between rows and 0.4 m apart between plants, planted in bags placed in gutters covered with 12-gauge plastic at their base. A density of 20,833 plants per hectare. A planting arrangement of 40 cm within-row spacing and 120 cm between-row spacing was employed. The soil surface was covered with 1.2-m-wide plastic mulch to prevent direct contact with the evaluated inoculum. A drip irrigation system was used to meet the water demand of each plant at various developmental stages and to prevent the spread of the pathogen. The nematode population in the soil used for planting was evaluated based on the number of second-stage juveniles (J2) using the centrifugation and flotation technique in a sugar solution [38], which also assessed the effectiveness of the disinfection process.

### 4.4. Obtaining the Eggs and Juveniles of Meloidogyne spp.

The extraction of *Meloidogyne* spp. eggs was carried out using the technique [38,39] from root samples of naturally infested tomato plants in the field. In the laboratory, the roots were washed, cut, placed in a bottle with 0.5% hypochlorite for 5 min with constant manual stirring, and then sieved (500, 146, and 25 µm). The sample retained on the 25 µm sieve was centrifuged at 3750 rpm for 5 min in 50 mL tubes, after which the supernatant was removed, followed by a second centrifugation with a sugar solution. The sugar was washed off using a 25 µm sieve. The eggs obtained were placed in artisanal sieves with filter paper on plastic plates following Whitehead’s technique [39] and incubated at 26 ± 2 °C in the dark for 14 days to obtain J2. The J2 were collected in a beaker, the sample was homogenized, and 1 mL of the solution was used to count individuals in a counting box with 42 grids under an Olympus^®^ CX31 microscope (Olympus corporation, Tokyo, Japan) with a 4X objective. This procedure was repeated three times to obtain the average J2 for each mL (initial concentration). The solution was adjusted to a final concentration of 1000 J2 in 50 mL of sterile distilled water [21], using the formula for dilutions and concentrations [40], and considering the initial volume, which contained the required volume according to the number of plants to be inoculated.

The pathogenic inoculum used in the study was sourced from Montelindo Farm. According to Cardona [41], *M. incognita* is the most prevalent species, determined through morphological and taxonomic characterization. Additionally, in 2022, based on molecular studies through amplification and sequencing of 28S (LSU) and 18S (SSU1 and SSU2) ribosomal subunits, that the inoculum affecting tomatoes on the same farm is composed mainly of 92% *M. incognita* and 8% of *M. arenaria* (unpublished data) [21].

### 4.5. Treatments with Chitosan

**Figure 1:**
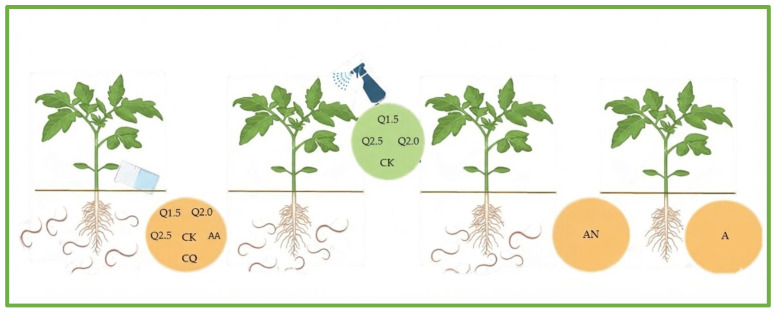
Description of treatments used in the study: Q1.5: chitosan, 1.5 mg/mL; Q2.0: chitosan, 2.0 mg/mL; Q2.5: chitosan, 2.5 mg/mL; CK: commercial chitosan control, 12.5 mg/mL; CQ: commercial chemical control (fluopyram), 2 cc/L; AA: acetic acid (1% and pH 5.6); A: control water without nematodes; AN: control water with nematodes. Created in Biorender and Microsoft Power Point. González, (2025) https://app.biorender.com/illustrations/65b5126e407a8784ece28449 (accessed on 13 December 2025).

Low-molecular-weight (50–190 kDa) chitosan from Sigma-Aldrich (Merck S.A., Darmstadt, Germany) was used for the evaluations, with a viscosity range of 20–100 centipoise and a degree of deacetylation between 75–85%. The study included eight treatments, consisting of three concentrations of low molecular weight chitosan (1.5, 2.0, and 2.5 mg/mL) and four control treatments applied to the soil or leaves as appropriate (Figure 1).

A stock solution of chitosan was prepared by dissolving 2 g of chitosan in 200 mL of a 1% acetic acid aqueous solution under constant agitation for 24 h. Subsequently, the pH was adjusted to 5.6 using KOH, and the solution was sterilized at 121 °C in an autoclave at 20 psi for 15 min [42].

It has been reported that chitosan molecule has a nematicidal effect [13,14,15,16,43]; however, acetic acid, used in the preparation of chitosan for its solubility, also has nematicidal effects [44,45,46]. Therefore, 1% acetic acid adjusted to the same pH was evaluated as a control to contrast these effects. The commercial controls used were fluopyram, a chemical recently used for nematode control [47,48], and ActiveChitosan, a 100% natural biostimulant made from plant extract and shrimp shell [49].

No foliar application of fluopyram was carried out, nor of the absolute controls (acetic acid and water with and without nematodes). Fluopyram, according to the label recommendations, must be applied to the soil, as done, who in a root penetration test showed that fluopyram only controlled the nematodes that remained in the rhizosphere [49]. Additionally, its withdrawal period is 100 days, so its application at the foliar level can be toxic. Its effect is direct contact with the nematode, inhibiting ATP biosynthesis [50]. Acetic acid can present phytotoxicity in plants, as it is effective and evaluated for its herbicidal effects [29,51,52], and its effect on nematodes has been evaluated in soil applications [27,52]. Water with and without nematodes was necessarily applied to the plant due to the parasitic nature and life habits of the nematode and the water requirements of the plant. Three applications were carried out over time with an interval of fifteen days according to the treatments. Necessary agronomic practices for plant health and nutrition were carried out [53].

### 4.6. Experimental Design

A completely randomized experimental design (CRD) was used, with a factorial arrangement (2 × 8) comprising two genotypes (IAC1687, LA2687) and eight treatments. The treatments included three concentrations of chitosan (1.5, 2.0, and 2.5 mg/mL), two commercial controls (ActiveChitosan and fluopyram), and three absolute controls (acetic acid and water with and without nematodes), with 50 cc per plant applied to the soil (edaphic application), resulting in 16 combinations with five repetitions per treatment. In contrast, foliar spray application of chitosan treatments (1.5, 2.0, and 2.5 mg/mL) and the commercial control (ActiveChitosan) was arranged in a completely randomized design among two genotypes and four treatments (2 × 4), totaling 8 combinations. For both types of application, the experimental unit was a plant (Figure 1). The first application was carried out after transplanting, when plants had developed the fourth true leaf. The second application was performed at the eight-leaf stage, and the third at the onset of flowering, approximately 30 days after transplanting.

### 4.7. Variables Evaluated

Production variables such as harvested fruit, average fruit weight, and yield per treatment (kg/ha) were evaluated. Final yield data were determined by extrapolating the mean fruit production per plant (g/plant) to kilograms per hectare (kg/ha). Based on the experimental planting design and spacing utilized, the established planting density was 20,833 plants per hectare. The conversion from production per plant (in grams) to yield per hectare (in kilograms) was performed using the following formula: Yield (kg/ha) = (Production/Plant (g)/1000 g/kg) × Planting Density (20,833 plants/ha). The resulting Production/Plant data were then subjected to statistical analysis.

Subsequently, at the end of the production cycle, destructive sampling of the plants was carried out to evaluate the number of eggs per 100 g of roots using technique [38]. The reproduction index (RI) was evaluated, which was calculated as the number of eggs per gram of tomato root with treatment application divided by the number of eggs per gram of roots of the control without treatment and with nematode inoculation (AN) multiplied by 100, following the methodology established by [54].

### 4.8. Statistical Analysis

The data obtained were subjected to analysis of variance (ANOVA), and the separation of main effects was performed using Tukey’s comparison tests at a 5% probability level, employing the SAS statistical program (version 9.4). The dataset was checked for normality, independence of errors, and homoscedasticity using the Shapiro–Wilk, Durbin–Watson, and Levene tests, respectively.

## 5. Conclusions

This work allowed us to understand the beneficial effect of chitosan on the yield components of the evaluated cultivars and its possible effect as a resistance inducer to reduce nematode populations, which, when applied to the soil or foliage, demonstrated its dual effects as a plant growth promoter and a controller of pathogenic nematodes. Chitosan can be a sustainable alternative in commercial production systems because it can help reduce the use of chemical pesticides and improve the health and productivity of crops.

The observed variability and imprecision in the nematode population data may be attributed to the use of acetic acid as the primary chitosan solvent, representing a significant confounding variable. This interference limited our ability to precisely determine the chitosan-specific mechanism of action. Consequently, we advocate that subsequent investigations prioritize the development or adoption of alternative solubilization protocols to isolate and accurately characterize the genuine biological effects of chitosan.

Formulations can combine acetic acid and chitosan to take advantage of their synergism, enhance nematicidal effects and promote plant growth and development. However, it is advisable when carrying out experiments with chitosan and its effect on microorganisms, to have acetic acid as a reference to compare the results and provide information on its effectiveness.

## Figures and Tables

**Table 1:** Yield response of cherry tomato IAC1687 and LA2076 (*Solanum lycopersicum* var. cerasiforme) and of the nematode population. A mixture of *Meloidogyne incognita* (92%) and *M. arenaria* (8%) to the edaphic application of chitosan.

Genotypes	Treatment	Production /Plant (g)	Yield (kg/ha)	Nematode Population (N° of Eggs 100 g of Roots)	Nematode Population (N° of Eggs 1 g of Roots)	Reproduction Index IR
IAC1687	Q1.5	635.2 c	13,233.1 c	10,670 b	106.7	40.59
	Q2.0	1145.3 ab	23,860.5 ab	5384 b	53.84	20.48
	Q2.5	1179.6 ab	24,575.8 ab	3898 b	38.98	14.83
	CK	1608.8 a	33,517.1 a	1842 b	18.42	7.01
	CQ	718.5 b	14,970.3 c	513 b	0.513	0.20
	AA	664.6 b	13,846.2 c	2647 b	26.47	10.07
	A	878.0 b	18,291.3 c	555 b	0.555	0.21
	AN	1093.8 b	22,787.5 ab	26,290 a	262.9	100
LA2076	Q1.5	917.9 b	19,124.3 b	9235 a	92.35	39.78
	Q2.0	816.4 b	17,010.1 b	7461 a	74.61	32.14
	Q2.5	1130.1 ab	23,545.0 ab	6810 a	68.1	29.33
	CK	1125.5 ab	23,448.9 ab	6163 a	61.63	26.55
	CQ	1033.3 ab	21,526.9 ab	853 a	0.853	0.37
	AA	821.1 b	17,106.2 b	3693 a	36.93	15.91
	A	1314.7 a	27,389.1 a	587 b	0.587	0.25
	AN	1397.7 a	29,118.9 a	23,215 a	232.15	100

Genotypes: IAC1687: accessions from the Agronomic Institute of Campinas (Brazil) and LA2076: accessions from the seed collection of the Tomato Resources Institute of California. USA. Treatment description (Treat): Q1.5: chitosan, 1.5 mg/mL; Q2.0: chitosan, 2.0 mg/mL; Q2.5: chitosan, 2.5 mg/mL; CK: commercial chitosan control, 12.5 mg/mL; CQ: commercial chemical control (fluopyram), 2 cc/L; AA: acetic acid (1% and pH 5.6); A: control water without nematodes; AN: control water with nematodes. Reproduction index (RI) = (Number of eggs per gram of root of each tomato genotype per treatment)/(Number of eggs per gram of root of the genotype without treatment and with nematode inoculation (AN)) × 100. Different letters denote statistical differences (*p* < 0.005).

**Table 2:** Yield response of cherry tomato IAC1687 and LA2076 (*Solanum lycopersicum* var. cerasiforme) and of the nematode population a mixture or *Meloidogyne incognita* (92%) and *M. arenaria* (8%) to the foliar application of chitosan.

Genotypes	Treatment	Production /Plant (g)	Yield (kg/ha)	Nematode Population (N° of Eggs 100 g of Roots)	Nematode Population (N° of Eggs 1 g of Roots)	Reproduction Index IR
IAC1687	Q1.5	1192.2 ab	24,837.7 ab	2223 c	22.23	8.46
	Q2.0	1287.7 a	26,827.3 a	3450 bc	34.5	13.12
	Q2.5	887.2 c	18,483.9 b	5269 ab	52.69	20.04
	CK	1124.4 ab	23,425.7 ab	7391 a	7391	28.11
LA2076	Q1.5	1416.1 a	29,503.3 a	4049 c	40.49	17.44
	Q2.0	1420.7 a	29,599.4 a	4524 bc	45.24	19.49
	Q2.5	977.9 b	20,373.6 b	7980 a	79.8	34.37
	CK	1231.6 ab	25,659.3 ab	7516 ab	75.16	32.38

Genotype: IAC1687: accessions from the Agronomic Institute of Campinas (Brazil) and LA2076: accessions from the seed collection of the Tomato Resources Institute of California. USA. Treatment description (Treat): Q1.5: chitosan, 1.5 mg/mL; Q2.0: chitosan, 2.0 mg/mL; Q2.5: chitosan, 2.5 mg/mL; CK: commercial chitosan control, 12.5 mg/mL. Reproduction index (RI) = (Number of eggs per gram of root of each tomato genotype per treatment)/(Number of eggs per gram of root of the genotype without treatment and with nematode inoculation (AN)) × 100. Different letters denote statistical differences (*p* < 0.005).

## Data Availability

The data are contained in the article.
